# Assessment of water ecosystem service value in the Jianghuai watershed area: a case study of Chuzhou

**DOI:** 10.1038/s41598-026-58818-y

**Published:** 2026-06-22

**Authors:** Ronghao Yang, Lizao Ye, Liming Xu, Donghua Chen, Xi Chen, Zice Ma, Xiulong Jin, Weixiang Kong, Wang Zhou

**Affiliations:** 1 School of Computer and Information Engineering, Chuzhou University, Chuzhou, 239000 China; 2 Anhui Province Key Laboratory of Realistic Geographic Environment, Chuzhou University, Chuzhou, 239000 Anhui China; 3 Anhui Engineering Laboratory of Geo-information Smart Sensing and Services, Chuzhou, 239000 Anhui Province China; 4 Anhui Center for Collaborative Innovation in Geographical Information Integration and Application, Chuzhou, 239000 Anhui China; 5 School of Literature and Media, Chuzhou University, Chuzhou, 239000 Anhui China

**Keywords:** Remote sensing, Water, Ecosystem service value, Spatiotemporal evolution, Jianghuai Watershed Area, Hydrology, Environmental impact

## Abstract

As a critical area for the ecological security barrier in the middle and lower reaches of the Yangtze River, the Jianghuai Watershed Area holds significant importance in coordinating ecological conservation and economic development through the assessment of its water ecosystem service value (ESV). This study focuses on Chuzhou, a typical city within the Jianghuai Watershed Area, to explore the spatiotemporal evolution of water ESV and analyze the spatiotemporal change of the Consistency between water Ecosystem services and Economy (CEE) from 2000 to 2022 based on remote sensing. Firstly, the water was extracted through normalized snow water index (NSWI) and Otsu threshold method (OTSU). Subsequently, the water ESV dynamic evaluation model was constructed. Using this model, the water ESV in Chuzhou was quantified, and its spatiotemporal distribution characteristics were analyzed. Finally, by calculating the coefficient of variation (CV) between ESV and GDP, the deviation between water ESV and GDP in Chuzhou was obtained. At the same time, the coordination between water ESV and economic development of Chuzhou was obtained by calculating CEE, so as to evaluate the Spatiotemporal coordination and regional differences between them. This study has found that: (1) The water ESV in Chuzhou showed a phased change of “fluctuating decline and subsequent recovery” from 2000 to 2022. (2) In terms of spatial distribution, the water ESV exhibits a gradient pattern of “high ESV in the northwest and low ESV in the southeast of the Chuzhou”. (3) In terms of ecological and economic coordination, the northwest of the Chuzhou maintain a healthy CEE. However, the southeast of Chuzhou has exacerbated the imbalance in CEE due to the water space occupied by construction land. This study could provide a decision-making basis for formulating ecological priority development strategies in the Jianghuai Watershed Area.

## Introduction

Water ecosystems, as critical carriers of freshwater resources and biodiversity, play irreplaceable roles in hydrological regulation, climate adjustment, and environmental purification^1,2^. However, under the dual pressures of global change and intensive human activities, the ecosystems are facing severe challenges: urban expansion and land-use conversion have led to the fragmentation and shrinkage of water bodies^3,4^. Agricultural intensification and industrial pollution have degraded water quality, while the imbalance between water ecological protection and economic development has further exacerbated the loss of water ecosystem service functions^5–7^. Quantifying the value of water ecosystem service value (ESV) has thus become a core tool for understanding the water ecological-economic balance, as it bridges the gap between water ecological functions and socioeconomic benefits^8^. Meanwhile, exploring the coordination between water ESV and economic growth (represented by GDP) is essential for breaking the “ecological degradation-economic inefficiency” trap and promoting regional sustainable development^9,10^.

The assessment of ESV has evolved from early global-scale estimations to localized, refined studies, with methodologies mainly falling into two categories: methods based on unit service function prices, which quantify value by evaluating economic benefits of specific services, and methods based on unit area value equivalent factors, which simplify assessment through standardized value coefficients per unit area Costanza et al^8,11^. laid the foundation by quantifying the global ecosystem service value, including aquatic systems^1^. Subsequent studies such as Cai et al. further optimized the evaluation framework by re-evaluating China’s ecosystem service value with more comprehensive factors^12^. Among these, Xie et al. improved the unit area equivalent factor method to adapt to China’s ecological context—it fully accounts for differences in ecosystem types and service functions across regions, features strong operability with clear calculation logic, and allows flexible adjustments based on local natural and socioeconomic conditions, thereby enhancing the accuracy and applicability of regional ESV assessments^8,11^. Recent studies have expanded into specific regions and ecosystems, such as wetland ESV evaluation in the Yellow River Delta^13^, freshwater supply service assessment in Jilin’s wetlands^3^, and dynamic analysis of ecosystem services in Japan’s national parks^14^, all highlighting the importance of localized adjustments^15^. However, existing research primarily focuses on large scales such as basins or provinces, with fewer studies exploring spatiotemporal heterogeneity at the county level—a scale critical for implementing precise ecological policies^16–19^.

For analyzing the coordination between ESV and GDP, common methods include coupling coordination degree models, spatial analysis tools, and consistency indices derived from spatial matching frameworks^9,20,21^. Coupling coordination degree models, as applied along the Silk Road Economic Belt in China to analyze aquatic ecosystem services and economic development interaction^21^, in subsequent studies of the Hohhot-Baotou-Ordos-Wuhai Urban Agglomeration to explore spatiotemporal dynamics of ESV and economy^22^, and in the Three Gorges Reservoir Area to investigate coupling with economic gravity center evolution^23^, quantify interaction intensity but often overlook spatial details^24^.Spatial analysis tools, such as the combination of spatial autocorrelation analysis and corresponding models, used to analyze the spatiotemporal evolution characteristics of the environmental and economic coordination index in Shaanxi Province^25^ and urban sustainability assessment in fast-growing cities^26^ capture local heterogeneity but require high-resolution data, limiting their county-level application^27^. Consistency indices, building on global studies integrating ESV and GDP spatially^28^ and regional analyses of localization-ecoenvironment coordination^29^, integrate spatiotemporal agglomeration of ESV and GDP, maintain clear links with administrative units, and allow flexible adjustment according to local characteristics^20,30^. This adaptability makes them well-suited for watershed areas with complex water systems, where capturing county-level dynamics between ecological services and economic development is critical^9,29^.

Chuzhou, located in the Jianghuai Watershed Area, is a representative region balancing ecological security and economic growth. As a core part of the Yangtze River Delta’s ecological barrier, it features a complex water network including natural lakes, rivers, and reservoirs, while undergoing rapid urbanization and industrialization. This dual identity makes it an ideal case for studying water ESV dynamics and ecological-economic coordination. This study aims to: (1) Extract water bodies using remote sensing data and the Normalized Snow Water Index (NSWI) combined with the Otsu threshold method; (2) Quantify the spatiotemporal evolution of water ESV in various counties, city and districts of Chuzhou from 2000 to 2022 using an improved unit area equivalent factor method; (3) Analyze the coordination between water ESV and GDP at the county level through a consistency index. The findings are expected to provide a scientific basis for ecological priority development in the Jianghuai Watershed Area.

## Materials and methods

### Study area

**Fig. 1:**
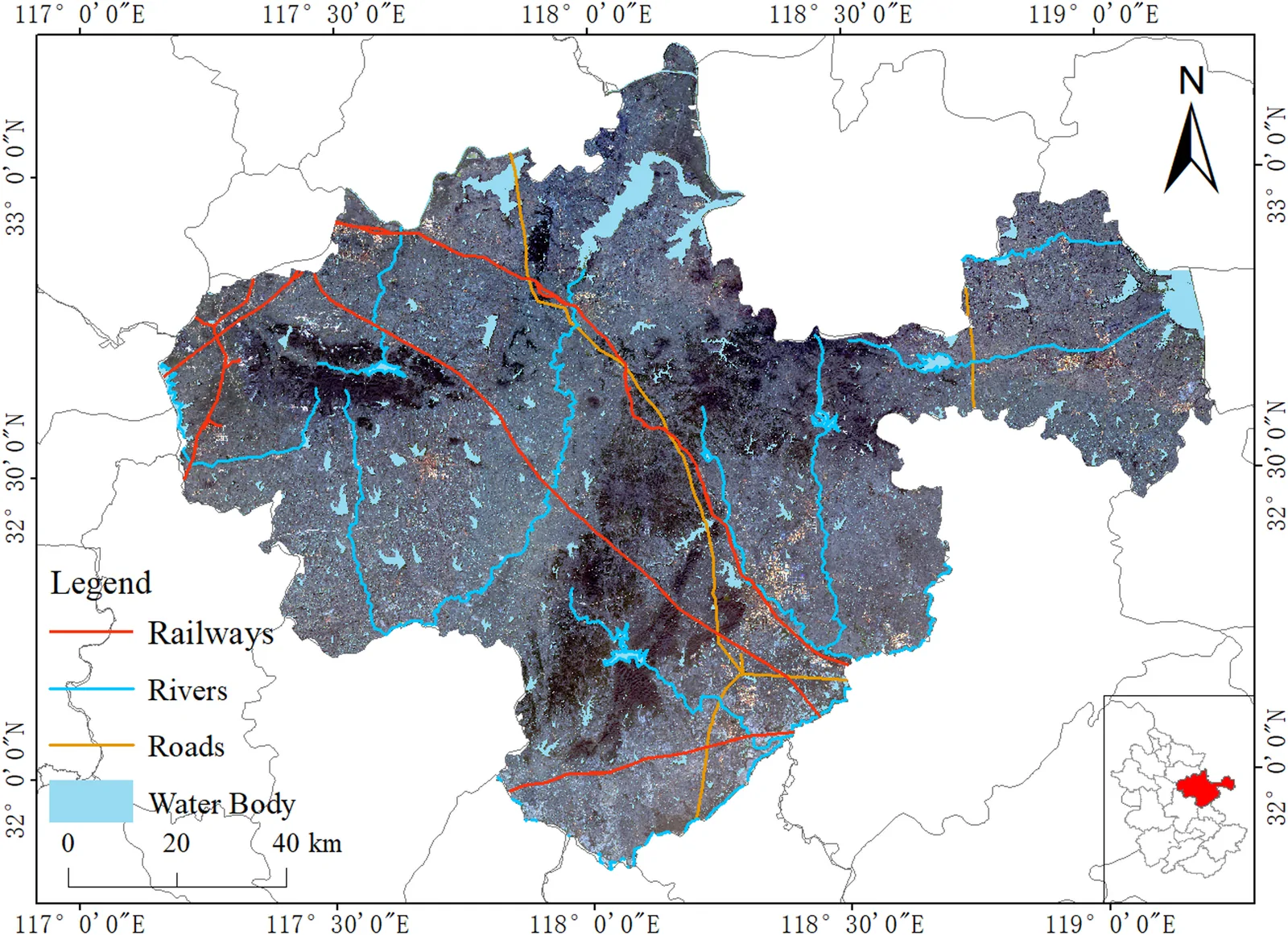
Overview of the study area. Using ArcMap 10.8.0. Software (URL: https://www.arcgis.com/home/index.html).

Chuzhou (117°09′E–119°13′E, 31°51′N–33°13′N) with a total area of 13,500 km², located in eastern Anhui Province, Chuzhou is a typical area in the Jianghuai Watershed Area. There are 8 research units in total (including 2 districts: Langya District(LY) and Nanqiao District(NQ), 4 counties: Fengyang County(FY), Dingyuan County(DY), Lai’an County(LA) and Quanjiao County(QJ), and 2 cities: Mingguang City(MG) and Tianchang City(TC)). The terrain is characterized by a northwest-high and southeast-low stepped distribution, including denuded hills, tablelands, and alluvial plains. The region has a typical water system belonging to the Huaihe and the Chuhe river basins, including natural lakes such as Gaoyou Lake and Nüshan Lake, as well as 437 artificial reservoirs, forming a composite water network. The overview of the research area is shown in Fig. 1. The climate exhibits both northern subtropical humid monsoon and warm temperate semi-humid characteristics, with an average annual temperature of 15.2 °C and precipitation of 1035 mm, showing uneven seasonal distribution and significant interannual variation. The vegetation coverage rate in the territory reaches 43.2%. It is distributed in seven types of water habitats, such as natural lakes, rivers and wetlands, and is a typical representative of the water land ecotone ecosystem in the middle and lower reaches of the Yangtze River Plain.

Socio-economically, Chuzhou has experienced significant economic growth from 2000 to 2022, with GDP data for each county and district showing a clear upward trend. For example, Tianchang City’s GDP increased from 40.9 billion yuan in 2000 to 686.11 billion yuan in 2022, while Langya District and Nanqiao District in the southeast also grew rapidly, with 2022 GDP reaching 250.69 billion yuan and 244.1 billion yuan respectively. As of 2022, the Gross Domestic Product (GDP) of Chuzhou City is 361 billion yuan, with a year-on-year increase of 5.5% calculated at comparable prices. Among them, the added value of the primary industry was 30.15 billion yuan, an increase of 3.7%; The added value of the secondary industry was 180.8 billion yuan, an increase of 7.7%; The added value of the tertiary industry was 150.05 billion yuan, an increase of 3.5%. The adjustment of the three industrial structures is 8.3:50.1:41.6, with industrial added value accounting for 40.1% of GDP. Urbanization and industrialization have driven notable land use changes, with construction land expansion concentrated in Langya and Nanqiao Districts, reflecting the interplay between economic development and land resources.

### Data sources

The data used in this study include administrative division, socioeconomic, and remote sensing image, with specific sources listed in Table 1. Socioeconomic data were collected and collated manually from various statistical yearbooks. Remote sensing image were projected into a unified geographic coordinate system using professional GIS software such as ENVI, and zonal statistics functions were applied to obtain values for each county and district.

**Table 1:** Introduction of data sources used in the study.

Data Type	Data Name	Data Source
Administrative Division	1:1 million public version basic geographic information data(2021)	National Catalog Service For Geographic Information (https://www.webmap.cn)
Socio-Economic	The total price of major grain crops and the total sowing The planting area of grain crops and the regional GDP of each county, city and district in Chuzhou	County Statistical Yearbook, Anhui Provincial Statistical Yearbook, City Statistical Yearbook, County Economic and Social Development Statistical Bulletin
Remote Sensing Image	Landsat 7 ETM+(2000–2012) image data GF-1 WFV(2013–2022) image data	science for a changing world(USGS) (https://earthexplorer.usgs.gov/) China Centre for Resources Satelite Data and Application (https://data.cresda.cn/#/home)

### Methods

#### Water information extraction

Considering the complex topographical conditions of Chuzhou and the characteristics of multi-source remote sensing image data, this study adopts a technical scheme combining the Normalized Snow Water Index (NSWI) and Otsu’s threshold method (OTSU) for water information extraction. The specific process is as follows: First, NSWI is calculated based on the multispectral bands of Landsat and GF-1 satellite images. This index enhances the spectral differences between water bodies and other land cover types, constructing an initial water feature space to lay the foundation for subsequent identification work. Subsequently, the OTSU algorithm is applied to perform adaptive threshold segmentation on the NSWI image, automatically determining the optimal segmentation threshold and generating candidate binary water extraction results. To address the misjudgment issues prone to occur in high-albedo areas and shadow regions, a spatial neighborhood statistical analysis method is further introduced to optimize the segmentation results. This effectively eliminates interference from non-water targets such as bare land, cloud-snow covered areas, and building shadows, ultimately generating complete vector data of water spatial distribution. In the subsequent sample-based accuracy verification, the extraction accuracy was confirmed to exceed 90%.

#### ESV assessment

The accounting of ESV can be roughly divided into two categories: methods based on unit service function prices and methods based on unit area value equivalent factors. Referencing the research of Xie and combining the actual situation of the study area, the unit area value equivalent factor method was used to account for the water ESV^8^. The study selected the average of the equivalent indices for water systems and wetlands from Xie’s revised equivalent factors of ESV per unit area in China as the water ESV equivalent for the study area^11^.The equivalent ESV per unit area in Chuzhou City is shown in Table 2. Based on the total price and sown area of major grain crops in Chuzhou, the economic value of food production services in farmland ecosystems per unit area was calculated using the following formula:

1 $$\:{E}_{a}=\frac{1}{7}\times\:\frac{{\sum\:}_{i=1}^{n}{N}_{i}}{{\sum\:}_{i=1}^{n}{M}_{i}}$$

where $\:{E}_{a}$ is the price of food production services in farmland ecosystems per unit area(yuan·hm⁻²), $\:{N}_{i}\:$ is the total price of grain crops in Chuzhou in year i(yuan), and $\:{M}_{i}\:$ is the sown area of grain crops in Chuzhou in year i(hm²). ﻿﻿Based on the revised water ESV per unit area in Chuzhou, the water ESV of Chuzhou was calculated using formula 2.

**Table 2:** Equivalent ESV per unit area in Chuzhou.

Service category	Single service	Value equivalent
Supply service	Food production	0.66
	Raw grain production	0.37
	Water supply	5.44
Adjustment service	Gas regulation	1.34
	Climate regulation	2.95
	Purify the environment	4.58
	Hydrological regulation	63.24
Expenditure service	Soil conservation	1.62
	Maintain nutrient cycling	0.13
	Biodiversity	5.21
Cultural service	Aesthetic landscape	3.31

2 $$\:ESV={\sum\:}_{k=1}^{n}{\sum\:}_{i=1}^{m}{\sum\:}_{j=1}^{l}\left({E}_{a}\times\:{E}_{k}\times\:{E}_{ij}\right)$$

where * ESV* is the total water ecosystem service value of the study area(yuan), $\:{E}_{k}$ is the equivalent of the k-th water ecosystem service function, and $\:{E}_{ij}$ is the area of the j-th water type in the i-th region(hm²).

#### CEE calculation

Quantifying the coordination between water ecosystem services and economic development is the key to achieving sustainable decision-making. Economic development at the municipal level is often reflected by GDP. This study referenced the Consistency of Population and Economy(CPE)^30^ to construct the Consistency of Ecosystem services and Economy(CEE) between water ESV and GDP in Chuzhou.

First, the Coefficient of Variation(CV) of water ESV and GDP was calculated to measure the degree of deviation between water ecosystem services and economic development levels, verifying the coordination between water ESV and GDP in the consistency model:

3 $$\:CV=\frac{1}{{Y}_{0}}\sqrt{\frac{{\sum\:}_{i=1}^{n}{\left({Y}_{i}-{Y}_{0}\right)}^{2}}{n}}$$

where $\:{Y}_{0}$ is the average value of water ESV/GDP in Chuzhou, n is the number of research units, and $\:{Y}_{i}$ is the water ESV/GDP value of the i-th research unit. A larger CV indicates greater differences in water ecosystem services or economic development.

Next, CEE was calculated to measure the coordination between water ESV and economic development in each research unit:

4 $$\:CE{E}_{i}=\frac{{{R}_{ESV}}_{i}}{{{R}_{GDP}}_{i}}=\frac{\frac{ES{V}_{i}}{{\sum\:}_{i=1}^{n}ES{V}_{i}}}{\frac{GD{P}_{i}}{{\sum\:}_{i=1}^{n}GD{P}_{i}}}$$

where $\:\:CE{E}_{i}$ is the consistency of ecosystem services and economic development for the i-th research unit, $\:{{R}_{ESV}}_{i}$ is the agglomeration level of ecosystem services in the i-th research unit, and $\:\:{{R}_{GDP}}_{i}$ is the agglomeration level of economic development in the i-th research unit.

Referencing the classification standard for the spatial consistency between population distribution and economic development in China^30^, when $\:CE{E}_{i}$ >1, it indicates that the agglomeration of ecosystem services in the region is higher than economic agglomeration; when $\:CE{E}_{i}$ < 1, it indicates that the agglomeration of ecosystem services is lower than economic agglomeration. The closer $\:CE{E}_{i}$ is to 1, the better the coordination between ecosystem services and economic development. Therefore, the coordination level between water ecosystem services and economic development in Chuzhou was divided into 3 major categories and 5 subcategories. The classification criteria for the coordination between ecosystem services and economic development are shown in Table 3.

**Table 3:** Classification criteria for the coordination between ecosystem services and economic development.

Classification Standard	Type	Subcategory
CEE ≤ 0.50 0.50 < CEE < 0.80	Economic agglomeration is higher than ecosystem service agglomeration.	Economic agglomeration is much higher than ecosystem service agglomeration. Economic agglomeration is higher than ecosystem service agglomeration.
0.80 ≤ CEE < 1.20	Ecosystem services and economic agglomeration are basically coordinated.	Ecosystem services and economic agglomeration are basically coordinated.
1.20 ≤ CEE < 2.00 CEE ≥ 2.00	Ecosystem service agglomeration is higher than economic agglomeration.	Ecosystem service agglomeration is higher than economic agglomeration. Ecosystem service agglomeration is much higher than economic agglomeration.

## Results

### Spatiotemporal heterogeneity of water ESV in Chouzhou

Based on the modified unit area equivalent factor method, the water ESV in each county, city and district of Chuzhou was obtained. Next, based on the obtained results, further analyze the spatiotemporal differentiation characteristics of water ESV in Chuzhou from 2000 to 2022.

#### Temporal evolution trend of water ESV

Temporally, the water ESV in each county and district of Chuzhou exhibited a complex dynamic change process. Overall, the total water ESV in the showed a trend of “fluctuating decline and subsequent recovery”. Before 2016, Chuzhou faced intense human activities. These included the expansion of cultivated land and the rapid occupation of water areas by construction land. Such activities caused the continuous reduction of water area. The structure and functions of the ecosystem were damaged. As a result, water ESV continued to decline. After 2016, the policy of returning farmland to wetlands was vigorously implemented. This led to a significant increase in the inflow of water area. The ecosystem was effectively restored, and water ESV gradually rebounded.

**Fig. 2:**
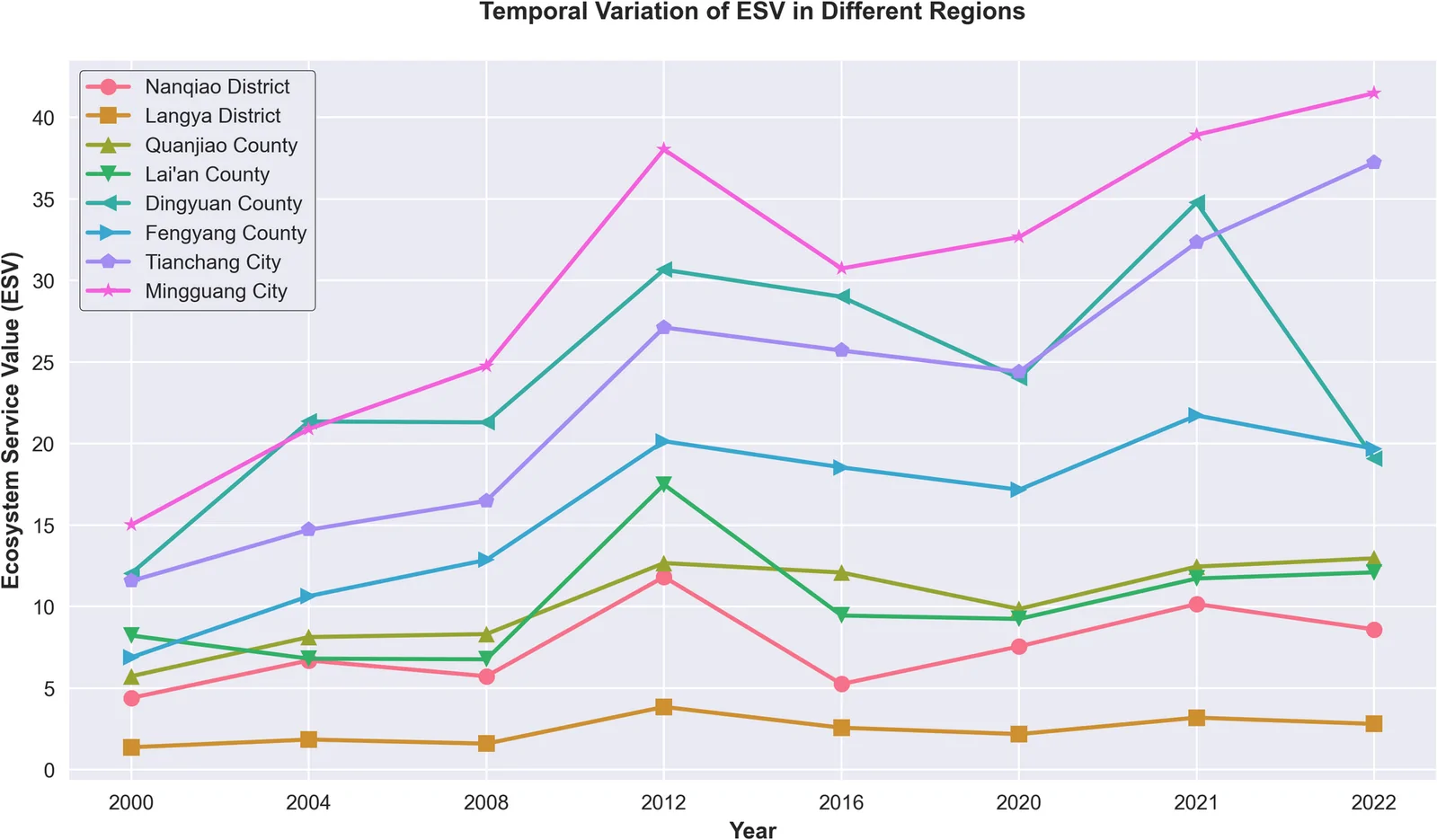
Water ESV variation curve in various counties, cities and districts of Chuzhou.

Specifically in each county and city, the water ESV in Nanqiao District and Langya District fluctuates sharply. This reflects the profound impact of frequent changes in land use types on water ecosystems in the urbanization process of the two regions; The water ESV of Quanjiao County showed an upward trend in the later stage. This indicates that the active and effective measures taken by the local area in ecological protection and water management have promoted the positive development of water ecosystems; The increase in water ESV in Lai’an County has tended to be flat after 2020, which may be due to various factors such as regional development models and resource and environmental constraints; The water ESV of Dingyuan County increased in the early stage and fluctuated significantly after 2016. This is closely related to the synergistic effects of regional land use policy adjustments, changes in economic development models, and climate change; The overall water ESV of Fengyang County, Tianchang City, and Mingguang City is on the rise. The growth trend of Mingguang City is particularly prominent. This indicates that these regions have taken a series of measures, including strengthening water ecological restoration and optimizing resource management. In the practice of ecological protection and sustainable development, these measures have played a positive role. They effectively enhance the service functions of water ecosystems and have achieved certain results in ecological construction in these areas. The trend of water ESV changes in various counties, cities and districts of Chuzhou is shown in Fig. 2.

#### Spatial differentiation pattern of water ESV

By using the natural breakpoint method, the water ESV of each research unit in Chuzhou was divided into five types.

From the perspective of spatial differentiation pattern, the water ESV in Chuzhou shows a significant “high water ESV in the northwest region and low water ESV in the southeast region” characteristic. Fengyang County and Mingguang City in the northwest are rich in water resources, with numerous lakes and wetlands. These water resources build a stable and functional ecosystem, laying the foundation for the efficient functioning of ecological services, therefore their water ESV is relatively high. Taking Fengyang County as an example, some lakes and wetlands within the county have rich biodiversity, which not only plays a key role in maintaining regional ecological balance, but also has important ecological functions such as regulating climate and purifying water quality, further enhancing regional water ESV.

**Fig. 3:**
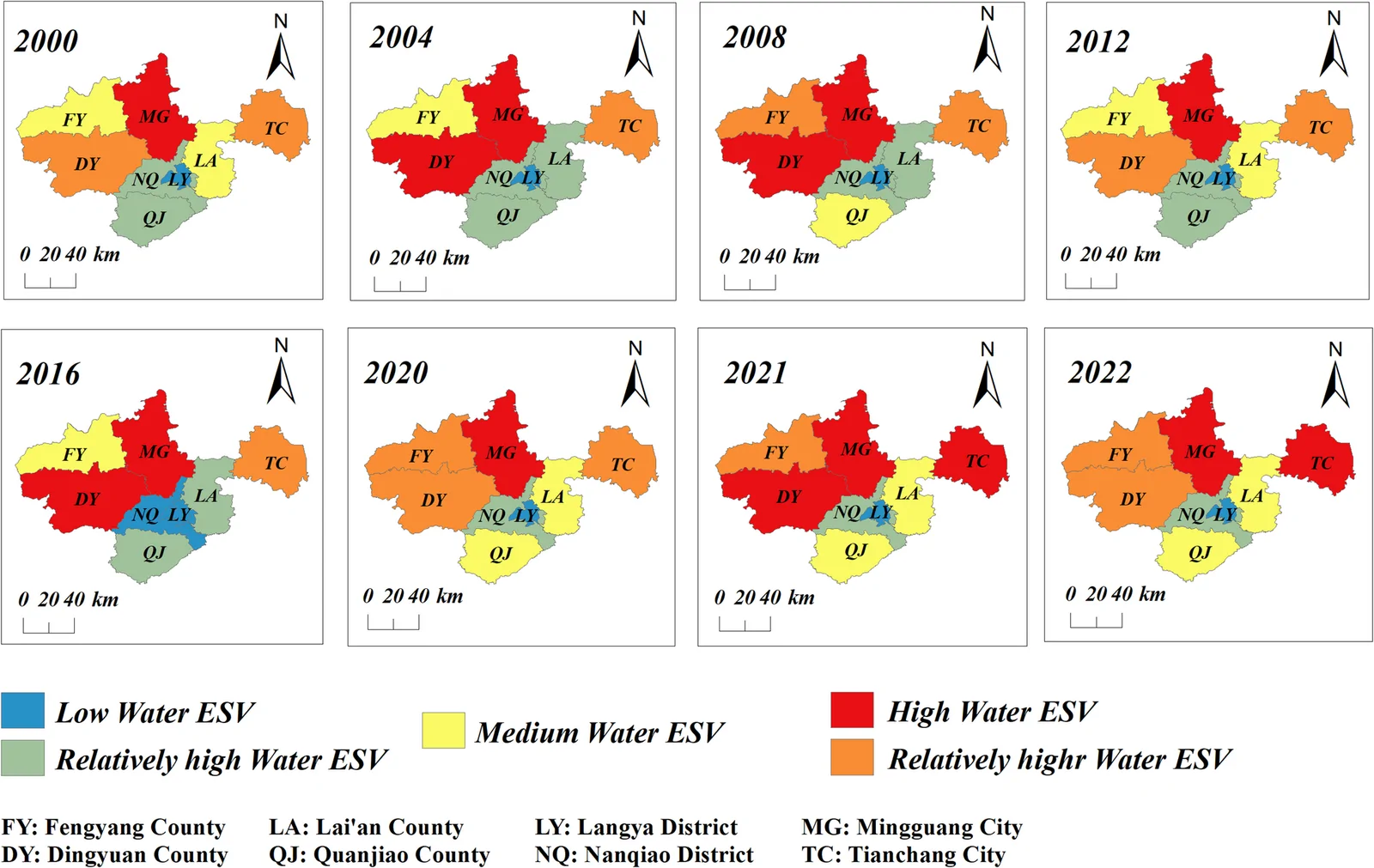
Spatial distribution of the water ESV in Chuzhou. Using ArcMap 10.8.0. Software (URL: https://www.arcgis.com/home/index.html).

The situation in Langya District and Nanqiao District in the southeast is completely different. The rapid urbanization process has driven large-scale expansion of construction land, and a large amount of water bodies have been encroached upon. This process seriously damages the ecosystem, significantly weakens ecological service functions, resulting in a significantly low water ESV and the formation of a “central collapse” phenomenon. The water ESV in Quanjiao County, Lai’an County, Dingyuan County, and Tianchang City is at an intermediate level. In different years, these regions have shown certain fluctuations in water ESV due to factors such as regional development strategies, implementation of ecological protection policies, and changes in the natural environment. In the process of balancing economic development and ecological protection in different regions, differences in development models and ecological protection measures ultimately lead to differences in the spatial distribution of water ESV. The spatial distribution of water ESV in Chuzhou City is shown in Fig. 3.

### Spatiotemporal CEE in Chuzhou

From the analysis of county, city, and district scales(Table 4), the coefficient of variation of water ESV in each region of Chuzhou is higher than that of GDP. This result indicates that compared to GDP, the regional differences in water ESV are more significant. However, through long-term observation of spatial distribution, it was found that the regional differences between the two did not show a significant trend of change over time. The coefficient of variation between water ESV and GDP in each county, city, and district is shown in Table 4.

**Table 4:** Coefficient of variation between water ESV and GDP in Chuzhou.

CV	Area	2000	2004	2008	2012	2016	2020	2021	2022
ESV	county	0.52	0.48	0.50	0.48	0.54	0.40	0.41	0.43
	city	0.23	0.28	0.36	0.21	0.21	0.24	0.21	0.26
	district	1.03	0.86	0.87	0.76	0.80	0.82	0.84	0.58
GDP	county	0.14	0.14	0.12	0.13	0.13	0.16	0.16	0.17
	city	0.09	0.16	0.46	0.55	0.59	0.53	0.56	0.58
	district	0.25	0.17	0.13	0.05	0.01	0.02	0.03	0.02

Note: The counties are Lai’an County, Quanjiao County, Dingyuan County and Fengyang County, the cities are Tianchang City and Mingguang City, and the districts are Nanqiao District and Langya District.

With reference to the classification criteria for the Coordination between Ecosystem Services and Economic Development, the standard differentiation is set up using GIS software. From the perspective of time evolution and spatial pattern.

#### Temporal evolution characteristics of CEE

From 2000 to 2012, the water CEE in Chuzhou’s counties, cities and districts underwent complex changes. In the early stage, CEE trended positively in some areas, with relatively balanced aggregation of ecosystem services and economic activities, marking a “coordination-dominated” phase. With the advancement of urbanization and industrialization, significant changes occurred in land use patterns across counties and districts, increasing pressure on the ecological environment. Take Nanqiao and Langya Districts as examples. Urban construction and industrial development in these areas required extensive land, leading to the encroachment of water areas for construction purposes. This process caused a decline in water ESV. Although their economies developed rapidly, the supply capacity of ecosystem services weakened, triggering fluctuations in eco-economic coordination and a gradual shift to a “game between ecosystem services and economic development” phase. Other regions like Quanjiao and Lai’an Counties also faced ecological protection challenges amid economic growth due to adjustments in development strategies and industrial structures, causing CEE values to fluctuate across years.

Between 2012 and 2016, the “game between ecosystem services and economic development” became more pronounced. As the economy further developed, the demand for land resources increased, intensifying conflicts between ecology and economy. In southeastern urban areas such as Langya and Nanqiao Districts, economic aggregation continued to rise, but insufficient attention to ecological protection led to a sustained decline in water ESV and a significant drop in CEE indices. This phenomenon highlights the mandatory impact of economic development on the environment development on the environment. Despite the implementation of ecological protection measures in some regions, accumulated ecological issues made it difficult to alleviate eco-economic conflicts in the short term.

From 2016 to 2022, while coordination in some areas fluctuated and rebounded, the overall “game between ecosystem services and economic development” remained evident. After 2016, policies such as returning farmland to wetlands were vigorously implemented, which effectively restored the water ecosystem in some regions, enhanced water ESV, and improved CEE. The mechanism through which these ecological restoration policies improve the regional water ecosystem involves three interrelated pathways:

First, by expanding water area. Returning farmland to wetlands directly converts previously cultivated land into water bodies or wetland habitats, increasing the total area of lakes, ponds, and riparian zones. This expansion enhances the water-holding capacity of the ecosystem, providing larger habitats for aquatic organisms and strengthening the base for maintaining biodiversity—a key component of water ESV as outlined in our assessment model^31,32^.

Second, by increasing vegetation coverage. The expanded water areas and restored wetland margins promote the growth of aquatic and semi-aquatic vegetation (e.g., Phragmites australis, Typha orientalis, and submerged macrophytes). These plants stabilize shorelines through their root systems, reducing soil erosion and sediment input into water bodies. Their canopies also intercept rainfall, slowing surface runoff and increasing infiltration, which regulates hydrological processes and supports groundwater recharge^33,34^.

Third, by reducing surface runoff and enhancing purification. The combined effect of expanded water areas and dense vegetation creates a more effective buffer against rainfall-runoff. Larger water bodies store more runoff, reducing flood peaks, while vegetation and wetland soils filter pollutants (e.g., nitrogen, phosphorus) from inflowing water. This dual role strengthens both hydrological regulation and water purification services, directly contributing to ESV recovery^35,36^.

**Fig. 4:**
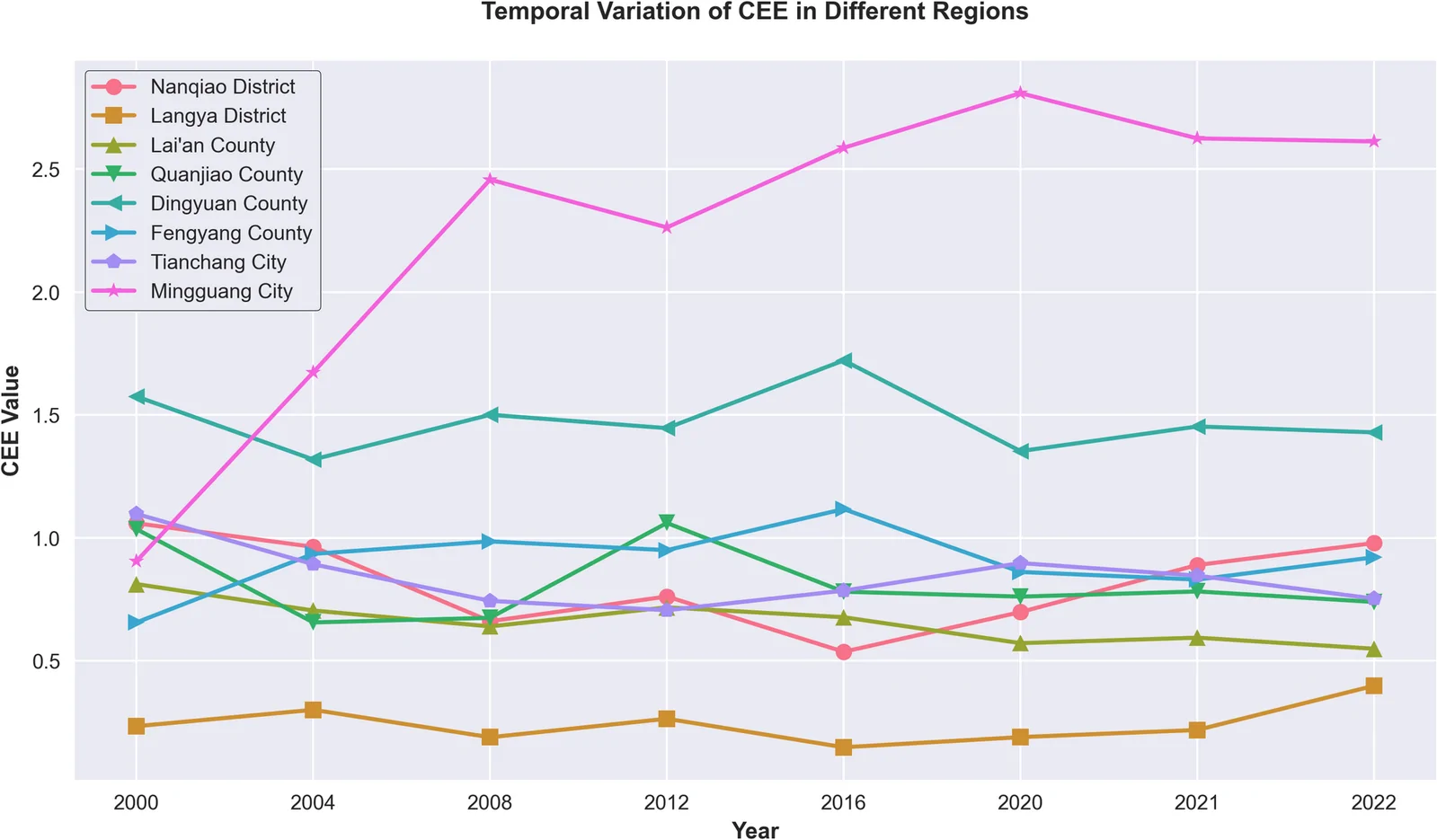
Change Curve of CEE in Various Counties, Cities and Districts of Chuzhou.

However, the cumulative damage to the environment from long-term economic development models, coupled with some areas’ continued over-reliance on ecological resources, meant that the growth rate of ESV still lagged far behind economic growth. The balance between ecosystems and economic development remained fragile, and the game between the two persisted. The trend of CEE values over time in each county, city, and district is shown in Fig. 4.

#### Spatial differentiation pattern of CEE

In terms of spatial distribution, significant regional differences exist in the water CEE in Chuzhou, presenting a pattern of “Regional sawing between ecological highlands and economic hotspots”. Fengyang County and Mingguang City in the northwest have built stable and functional ecosystems with abundant lakes, wetlands and other water resources, leading to high water ESV. During economic development, the two regions have well balanced the relationship between economic growth and ecological protection. Through measures such as reasonable land use planning and strengthened ecological environment governance, they have continuously exerted ecosystem service functions, maintaining relatively high CEE. For example, Fengyang County has actively developed ecological agriculture and eco-tourism, protecting the water ecological environment while promoting economic growth, thus maintaining a high CEE.

As economic development hotspots, Langya District and Nanqiao District in the southeast have experienced rapid urbanization and industrialization, with extensive expansion of construction land. A large number of water areas have been converted into urban construction land and industrial land, resulting in a significant reduction in water area, severe damage to aquatic ecosystems, and a notable decline in water ESV. These regions show a high degree of economic agglomeration, but the supply of ecosystem services is insufficient, leading to prominent contradictions in the coordination between ecosystem services and economic development and a low CEE.

The lack of reasonable protection and planning of the aquatic ecosystem in these areas has weakened its regulation, support, and cultural functions through specific pathways.

For regulation functions, uncontrolled occupation of water areas has fragmented the original connected water network, reducing hydrological connectivity. This fragmentation impairs the ecosystem’s capacity for hydrological regulation: the loss of wetlands and lakes, which play a key role in storing excess rainfall, weakens flood mitigation capabilities. Meanwhile, the reduction in water surface area affects local microclimate regulation, as the decreased evaporation from water bodies alters temperature and humidity patterns. These changes directly weaken the “hydrological regulation” and “climate regulation” services, which are important components of water ESV^37,38^.

In terms of support functions, the reduction in water area and destruction of aquatic habitats disrupt the stability of aquatic ecosystems, leading to a decline in aquatic biodiversity. This loss of species affects the ecosystem’s ability to maintain nutrient cycling, as key organisms involved in processes such as nitrogen and phosphorus transformation are reduced. Additionally, the degradation of riparian vegetation due to unplanned development increases soil erosion, leading to increased sediment input into water bodies and further deterioration of water quality. These impacts compromise the “biodiversity maintenance” and “nutrient cycling” services, which are critical to the support function of the ecosystem^39,40^.

Regarding cultural functions, the destruction of natural water landscapes, such as the conversion of riverside wetlands and lakeshores into built-up areas, reduces the aesthetic and recreational value of aquatic ecosystems. This diminishes opportunities for public engagement with natural water environments, affecting eco-tourism and the provision of spiritual and cultural benefits. Consequently, the “cultural service” function, which contributes to the overall value of the ecosystem, is weakened^41,42^.

The failure to integrate aquatic ecosystem protection into land-use planning has thus disrupted the multi-functional balance of the ecosystem, creating a mismatch between economic development and the supply of ecological services in southeastern Chuzhou.

Regions such as Quanjiao County, Laian County and Tianchang City have moderate coordination levels with certain fluctuations in different years. These regions face both economic growth pressure and ecological protection requirements during economic development.

**Fig. 5:**
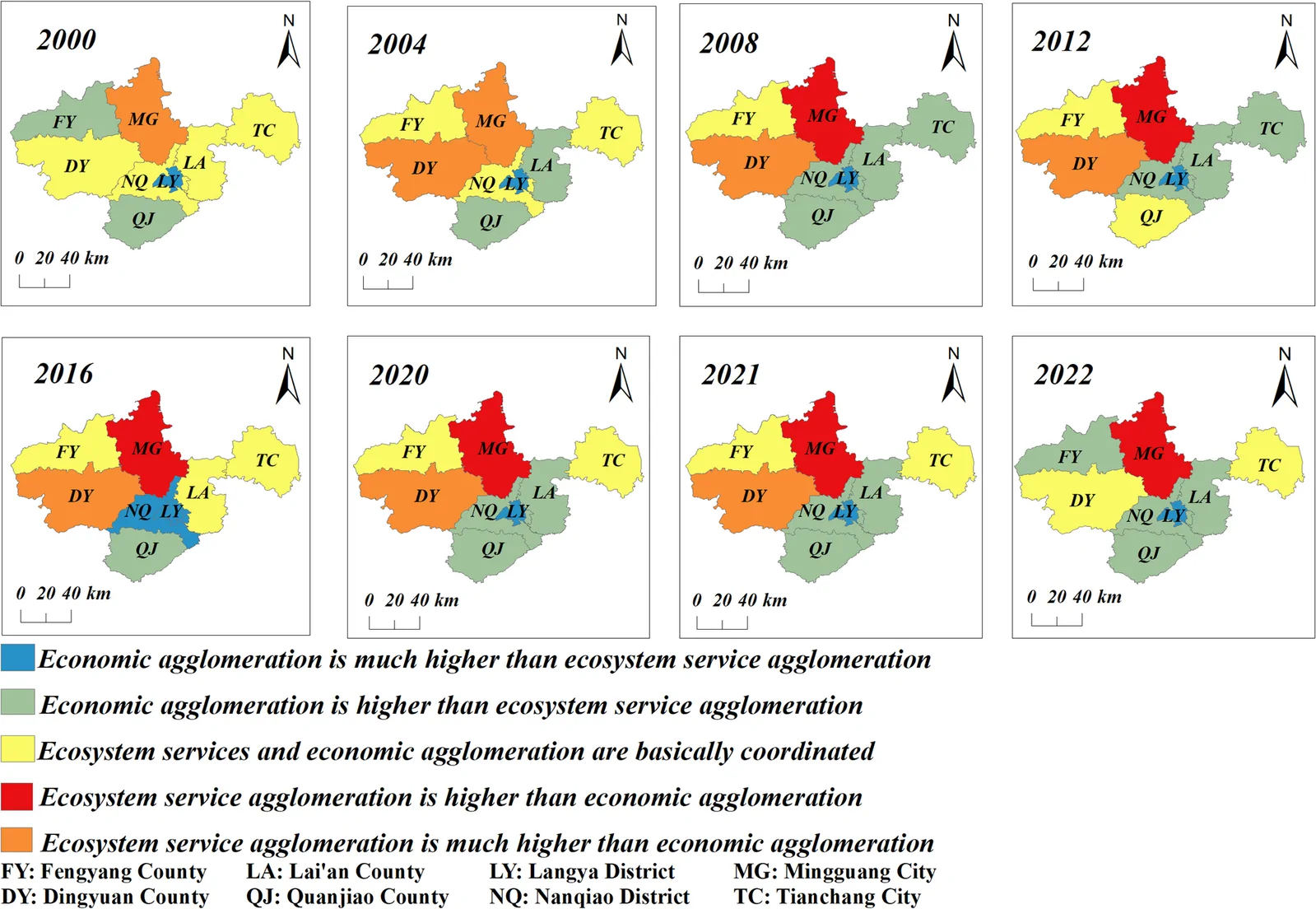
Spatial distribution of the CEE in Chuzhou. Using ArcMap 10.8.0. Software (URL: https://www.arcgis.com/home/index.html).

In Laian County, after 2008, undertaking industrial expansion led to relatively lagging ecological protection investment, causing coordination to fluctuate. after 2016, with the advancement of policies such as returning farmland to wetlands, local governments strengthened wetland restoration and pollution control, and coordination gradually developed in a positive direction. Quanjiao County’s coordination slightly increased during 2004–2008 when promoting agricultural ecological transformation, but decreased slightly around 2012 due to the impact of rapid construction land expansion; after the implementation of ecological compensation policies in 2016, coordination stabilized as ESV increased. Such fluctuations intuitively reflect the game between “economic growth orientation” and “ecological protection investment” in different development stages. Around 2012, some regions experienced a downturn in the coordinated development of ecology and economy in pursuit of short-term economic growth. During this period, the coordination between the two significantly decreased, resulting in the phenomenon of the so-called “watershed value” trough. In 2016, with the implementation of the 13th Five Year Plan for ecological planning, the situation began to turn around. Various regions have gradually improved the ecological environment through a series of measures such as restoring blue-green spaces and optimizing industrial structures. During this process, the interaction between ecology and economy gradually shifted from contradiction to synergy. This change fully confirms that policies play a crucial driving role in regulating the coordinated relationship between ecosystem services and economic development. The spatial distribution of CEE in Chuzhou City is shown in Fig. 5.

## Discussion

This study reveals the spatiotemporal evolution law of water ESV in the Jianghuai Watershed Area and its synergy mechanism with economic development through multi-source data integration and dynamic model analysis. Discussions are conducted from three aspects: driving mechanisms, methodological significance, and practical implications.

The implementation of the returning farmland to wetlands policy significantly promoted the recovery of water ESV, but the spatial differentiation of its effects revealed the complexity of ecological restoration. In the northwest region with abundant natural lakes and wetlands, the complete ecological background led to a positive feedback between improved water connectivity and vegetation recovery after policy implementation, accelerating ESV recovery. In the southeast, historical development accumulation effects caused reduced ecological recovery thresholds due to water fragmentation and hardened surface expansion, leading to diminishing marginal benefits of policy intervention. This result is consistent with the “ecological restoration window period” theory proposed in existing research, indicating that more proactive ecological space reconstruction measures are needed in highly urbanized areas^43–46^.

The “high value in the northwest and low value in the southeast” gradient pattern of water ESV arises from the interplay of natural geography and human activity. The hilly-lake landscape in the northwest sustains stable ecological services through enhanced precipitation retention, while rapid urbanization in the southeast disrupts hydrological regulation via water area occupation and construction land expansion. Reduced water area diminishes natural storage capacity, lowering groundwater recharge and accelerating surface runoff—consistent with McGrane’s observation that urban water cycle disruption begins with lost hydrological buffering^47^. Concurrently, impervious construction surfaces exacerbate these effects. Oswald et al. noted such surfaces reduce infiltration and evapotranspiration, while Du et al. quantified their role in increasing flood peaks through concentrated runoff^48,49^. This dual impact—water loss and impervious expansion—weakens microclimate regulation and pollutant purification, directly impairing regulatory services. This spatial pattern aligns with landscape ecology’s “pattern-process-service” framework, underscoring the need to balance ecological preservation and development.

The dual pattern of coordination between ecosystem services and economic development reflects the institutional locking effect of regional development paths. Through the ecological compensation mechanism and green industry cultivation, the Chuzhou’s northwest has realized the internalization of ecological protection costs and promoted the evolution of CEE index to a balanced state; However, affected by the traditional GDP assessment orientation, the priority strategy of land development in the Chuzhou’s southeast has led to the decoupling of ecological service supply and economic development demand, falling into a negative cycle of “ecological degradation and increased compensation input”. This discovery provides a new perspective for solving the “dilemma of balancing ecology and economy”. That is, the ecological value needs to be incorporated into the regional development cost accounting system through institutional innovation.

This study constructs a water ESV assessment framework suitable by integrating remote sensing dynamic monitoring and modified equivalent factor methods. And its regional governance orientation provides methodological reference for similar studies. However, there are limitations in quantifying socioeconomic driving factors such as policy implementation intensity and market mechanisms. In the future, multi-agent game models should be combined to deepen mechanism analysis. Additionally, the quantification accuracy of cultural service functions in ecological service value assessment needs improvement, and new data sources such as social media data can be introduced for supplementation.

## Conclusions

This study comprehensively analyzed the spatiotemporal evolution law of water ESV in Chuzhou and its synergy with economic development. The main conclusions are as follows:

From 2000 to 2022, the water ESV in Chuzhou showed a phased evolution trend of “fluctuating decline and subsequent recovery”. Before 2016, the water ESV showed a fluctuating downward trend; after 2016, ESV recovered. The spatial distribution of water ESV in Chuzhou exhibited a gradient difference of “High value agglomeration in northwest and low value collapse in southeast of the Chuzhou”. Due to the high integrity of the ecosystem of lakes and wetlands, the water ESV in the northwest of Chuzhou is significantly higher than that in the southeast of Chuzhou with the highly urbanized. The water ESV of Fengyang County and Mingguang City in the northwest is significantly higher than that of other regions. Furthermore, through reasonable resource management, these two regions have maintained a virtuous coordination between ecological services and economic development. Langya District and Nanqiao District in the southeast form a “low value center” of water ESV. The level of ecological service agglomeration is far lower than that of economic agglomeration, and the imbalance of regional CEE is intensifying. This phenomenon highlights the contradiction between ecological protection and economic development in the process of rapid urbanization. At the same time, it also reflects the differentiated impact of natural endowments and human activity intensity on the regional water ESV.

## Data Availability

The datasets used and analyzed during the current study are available from the corresponding author on reasonable request.
